# Development of Radiolabeled Compounds for Molecular Imaging and Imaging-Based Therapy

**DOI:** 10.1155/2015/365418

**Published:** 2015-03-26

**Authors:** Kazuma Ogawa, Masahiro Ono, Mei Tian, Masashi Ueda, Takahiro Higuchi

**Affiliations:** ^1^Graduate School of Medical Sciences, Kanazawa University, Kanazawa 920-1192, Japan; ^2^Graduate School of Pharmaceutical Sciences, Kyoto University, Kyoto 606-8501, Japan; ^3^Department of Nuclear Medicine and Medical PET Center, The Second Hospital of Zhejiang University School of Medicine, Zhejiang, China; ^4^Graduate School of Medicine, Dentistry and Pharmaceutical Sciences, Okayama University, Okayama 700-8530, Japan; ^5^Molecular and Cellular Imaging, Comprehensive Heart Failure Center, Universitätsklinikum Würzburg, Würzburg 97078, Germany

In recent years, a companion diagnosis for prediction of the effectiveness and the side effects after administration of drugs has become very important to decide a therapeutic strategy. In USA, Food and Drug Administration (FDA) recommends the development of the companion diagnostic agent simultaneously with a new drug development. Nuclear medicine imaging using positron emission tomography (PET) or single photon emission computed tomography (SPECT), which visualizes target molecules, is able to detect biological and biochemical changes in the earlier phases of diseases differing from an anatomical imaging using X-ray computed tomography (X-ray CT) or magnetic resonance imaging (MRI). Radiolabeled tracers for the nuclear medicine have great potential to provide a lot of information about pathology of individual patients depending on characteristics of each tracer and could be the companion diagnostics. Accordingly, molecular imaging and imaging-based therapy using radiolabeled tracers must contribute to the realization of personalized medicine.

11 selected papers are published in this special issue. Most of them deal with important topics in nuclear medicine. Below is a brief introduction to some of these papers in this special issue.

In cerebral imaging, imaging of tau protein is useful for presymptomatic diagnosis and monitoring of the progression of Alzheimer's disease because the presence of neurofibrillary tangles, composed of hyperphosphorylated tau protein, is well correlated with neurodegeneration and cognitive decline in Alzheimer's disease. H. Watanabe et al. summarized tau imaging probes for PET and SPECT.

In oncology, molecular targeted therapies using drugs, such as tyrosine kinase inhibitors and monoclonal antibodies, become the mainstream in cancer therapy. It is important to estimate the target molecule status in cancer tissues and predict therapeutic efficacy. M. Yoshimoto et al. introduced new radiolabeled tyrosine kinase inhibitors and antibodies for theragnostic imaging and their clinical application in molecular targeted therapy.

In the point of view from early detection of response to therapy, apoptosis imaging could be useful because apoptosis occur before anatomical change at posttreatment and could contribute to personalized medicine. K. Ogawa and M. Aoki introduced radiolabeled compounds for molecular imaging of apoptosis (cell death), their applications, and utility in medicine.

Hypoxic regions in tumors are known to be resistant to chemotherapy and radiotherapy. Hypoxia-inducible factor-1 (HIF-1) expressed in hypoxic regions regulates expression of genes related to tumor growth, angiogenesis, metastasis, and therapy resistance. Thus, imaging of HIF-1-active regions in tumors is of great interest. M. Ueda and H. Saji summarized the current status of HIF-1 imaging in preclinical and clinical studies.

*Kazuma Ogawa* *Kazuma Ogawa* *Masahiro Ono* *Masahiro Ono* *Mei Tian* *Mei Tian* *Masashi Ueda* *Masashi Ueda* *Takahiro Higuchi* *Takahiro Higuchi*

